# Measurement of factor XIII for the diagnosis and management of deficiencies: insights from a retrospective review of 10 years of data on consecutive samples and patients

**DOI:** 10.1016/j.rpth.2025.102689

**Published:** 2025-01-23

**Authors:** Mohammed Abdullah Al Sharif, Natalie Mathews, Subia Tasneem, Karen A. Moffat, Stephen A. Carlino, Siraj Mithoowani, Catherine P.M. Hayward

**Affiliations:** 1 Department of Pathology and Molecular Medicine, McMaster University, Hamilton, Ontario, Canada; 2 Department of Pathology and Laboratory Medicine, King Abdullah Bin Abdulaziz University Hospital, Princess Nourah University, Riyadh, Saudi Arabia; 3 Division of Hematology-Oncology, Department of Pediatrics, Centre Hospitalier Universitaire Sainte-Justine, Université de Montréal, Montréal, Québec, Canada; 4 Department of Medicine, McMaster University, Hamilton, Ontario, Canada; 5 Special Coagulation, Hamilton Regional Laboratory Medicine Program, Hamilton, Ontario, Canada

**Keywords:** blood coagulation disorders, blood coagulation factor inhibitors, blood coagulation tests, factor XIII deficiency, factor XIII, hemostatic disorders, inherited coagulation disorders

## Abstract

**Background:**

Factor XIII (FXIII) deficiency is a challenge in the diagnosis of rare bleeding disorders with inherited and acquired causes.

**Objectives:**

We evaluated consecutive cases tested for FXIII deficiency for insights on diagnosis.

**Methods:**

With ethics approval, we retrospectively reviewed FXIII tests performed between 2013 and 2023 and local patient records for insights into causes and presentations of FXIII deficiency.

**Results:**

Two thousand one hundred ninety-one samples from 1915 patients (ages: 0-90 years; 38% local) were tested. The FXIII activity (FXIII:Act; Berichrom FXIII, Siemens Healthcare) was low in 14%/9.7% of tested samples/patients. FXIII subunit A antigen (FXIII-A:Ag; Werfen HemosIL FXIII antigen; low in 45% of 251 samples) helped characterize FXIII deficiency severity and identify type 2 deficiencies from acquired FXIII inhibitors. Urea clot solubility tests (18.2% requested without FXIII:Act) were largely noninformative as all abnormal samples (*n* = 7) had undetectable FXIII-A:Ag levels. Excluding FXIII inhibitor patients, FXIII:Act showed strong correlation with FXIII-A:Ag (*R*^2^ = 0.84, *P* < .001) and weak correlation with plasma fibrinogen (*R*^2^ = .005, *P* < .001). Some patients had combined acquired FXIII and fibrinogen deficiencies from consumption or major bleeding. FXIII-deficient and nondeficient patients had similar bleeding except for more umbilical and gastrointestinal bleeding among deficient patients (*P* < .05). Most FXIII deficiencies were acquired (92%), and although several were autoimmune, most were from consumption, major bleeds, or severe infections or had uncertain significance, with bleeding sometimes attributable to other causes.

**Conclusion:**

Congenital and acquired FXIII deficiency are associated with bleeding. Local practices were changed to ensure that FXIII:Act is used to screen for FXIII deficiency and that deficient patients have FXIII:Act and FXIII-A:Ag quantified and compared.

## Essentials

• Congenital and acquired factor XIII (FXIII) deficiency are important causes of bleeding.
• We retrospectively assessed the prevalence, findings and causes of FXIII deficiency.
• FXIII deficiency was found in 14.1%/9.7% of samples/patients, with 92% from acquired causes.
• Most evaluated FXIII-deficient and nondeficient patients had bleeding.

## 1 Introduction

Blood coagulation factor XIII (FXIII) is an important protein that crosslinks and stabilizes fibrin, making fibrin more resistant to fibrinolysis [1]. FXIII circulates as a protransglutaminase, requiring activation by thrombin, with fibrin serving as the cofactor for activated FXIII (FXIIIa) [1]. Plasma FXIII is synthesized in the liver, forming FXIII-A_2_B_2_ heterodimers that contain 2 catalytic FXIII-A subunits (encoded by *F13A1*) and 2 carrier FXIII-B subunits (encoded by *F13B*) that prolong the half-life of plasma FXIII [1]. Platelet FXIII (which is made by megakaryocytes) lacks FXIII-B_2_ and promotes clot retraction and stability [1,2]. In plasma, FXIII-A_2_B_2_ and free XIII-B_2_ are bound to fibrinogen via fibrinogen γ chain residues 390 to 396 that mediate fibrin’s FXIIIa cofactor function [3]. Relationships between plasma FXIII and fibrinogen levels have not been widely explored. Among patients undergoing cardiac surgery, FXIII levels show correlation to fibrinogen levels and rotational thromboelastometry estimates of fibrin contributions to clot firmness [4].

As FXIII deficiency is rare, the diagnosis requires clinical suspicion, particularly when coagulation tests do not reveal an alternative cause for serious bleeding [5]. Congenital FXIII deficiency affects ∼1:1 to 4 million people worldwide and is more prevalent in regions in which consanguinity is common [1,6,7]. *F13A1* mutations are the most common cause and result in more severe deficiencies and bleeding than *F13B* mutations, which result in a loss of FXIII-B carrier function [1,6,7]. Carriers often have milder, asymptomatic FXIII deficiency [1,6,7]. Severe FXIII deficiency typically presents with spontaneous bleeding that can include intracranial hemorrhage (ICH), umbilical cord bleeding, delayed challenge-related bleeding (∼12-36 hours after hemostatic challenges), impaired wound healing, and pregnancy loss [1,6,8]. FXIII replacement therapy (which increases plasma but not platelet FXIII) dramatically improves outcomes [1]. Data for treated severe FXIII-deficient patients suggest that FXIII levels of ≥0.31 U/mL (or 31%, 95% CI estimates: ∼0.11-0.51 U/mL) result in complete bleed prevention, with significantly more bleeds below 0.15 U/mL [8].

Acquired FXIII deficiency is a rare and sometimes fatal cause of bleeding, with immune and nonimmune causes [1,9,10]. Acquired deficiencies from autoantibodies can inhibit FXIII function and/or accelerate FXIII clearance [10]. Acquired nonimmune FXIII deficiencies are associated with high rates of bleeding and prolonged intensive care unit (ICU) stays [11]. Among ICU patients, an FXIII activity of <0.50 U/mL is associated with receiving more red cell transfusions [12]. Acquired FXIII deficiency has been associated with trauma, major bleeding, infection, burns, and other nonimmune causes, and there are controversies about the levels that merit FXIII replacement [13].

The diagnosis of congenital and acquired FXIII deficiency remains challenging [1]. The International Society on Thrombosis and Haemostasis (ISTH) recommends quantitative FXIII activity and antigen assays and additional tests not performed in clinical laboratories (eg, platelet lysate FXIII assessment) [1]. Simplified algorithms recommend quantifying FXIII activity and FXIII antigen, evaluating for inhibitors and considering genetic testing, if available [7]. While quantitative FXIII antigen and activity assays are available in some regions, including Europe, the only Food and Drug Administration–approved diagnostic FXIII assay in the United States is the HemosIL FXIII antigen (Werfen), which quantifies FXIII-A:Ag, and the only Health Canada–approved FXIII assays are the HemosIL FXIII antigen and the Berichrom FXIII assay (Siemens Healthcare; abbreviated as FXIII:Act). The HemosIL FXIII antigen and the Berichrom FXIII assay are the most widely used kits [14,15]. In resource-challenged countries, access to appropriate FXIII diagnostic assays is a significant problem [7].

While FXIII clot solubility assays are simple, inexpensive tests that many laboratories continue to perform, they only detect very severe FXIII deficiencies (levels < 0.02 U/mL) [1,14,15]. Quantitative FXIII:Act and FXIII:Ag assays are the most helpful to diagnose and monitor severe and milder deficiencies [1], including those implicated in postoperative and ICU bleeds [[11], [12], [13]]. As few laboratories perform quantitative FXIII assays, many send samples to a reference center (such as our own laboratory). Plasma FXIII-A antigen (FXIII-A:Ag) and FXIII:Act levels are expected to show strong correlation, as both quantify FXIII enzymatic subunit [16].

Current quantitative FXIII:Act assays measure either FXIIIa-catalyzed release of ammonia from FXIII substrates (eg, Berichrom FXIII; Technochrom FXIII kit—a research use only kit from Technoclone) or labeled amine incorporation into protein substrates (eg, Technofluor FXIII Act assay, Technoclone) [1]. To accurately quantify FXIII:Act by ammonia release assays, particularly in the low range (eg, <0.14-0.20 U/mL), it is recommended to run each sample with an iodoacetamide blank [1,17], which is included in the Technochrom FXIII kit but not the Berichrom FXIII assay. Despite these recommendations, many diagnostic laboratories perform the Berichrom FXIII assay unmodified, without an iodoacetamide blank [14,15], due to safety restrictions on iodoacetamide use and/or concerns about modifying assays to create “laboratory-developed tests.”

Several organizations have made efforts to increase physician awareness of FXIII deficiency, which can cause life-threatening bleeding [10,18]. Nonetheless, some physicians are unaware that quantitative FXIII:Act tests are recommended for the diagnosis and lack information on common causes of FXIII deficiency, and their association with bleeding.

Our center has offered quantitative FXIII:Act and FXIII-A:Ag tests, FXIII inhibitor tests, and factor XIII clot solubility assays for patients across Canada for many years. We initiated a retrospective review of the past decade of findings for consecutively tested clinical samples (local and external) and medical record review of local cases to gain unbiased insights into the findings, causes, and presentations of congenital and acquired FXIII deficiencies and if their bleeding differs from tested patients without FXIII deficiency. Our secondary goals were to explore 1) relationships between FXIII activity and antigen and conditions that reduce FXIII activity more than FXIII antigen, 2) potential relationships between plasma fibrinogen and FXIII:Act for local patients given that plasma FXIII circulates bound to fibrinogen, and 3) opportunities to further improve FXIII test utilization and reporting, considering recent, simplified testing algorithms [7].

## 2 Methods

The retrospective study was approved by the Hamilton Integrated Research Ethics Board (project 16804). Briefly, the Hamilton Regional Laboratory Medicine Program (HRLMP) electronic records for 2013 to 2023 were queried with the goal of evaluating ≥200 representative, abnormal FXIII activity results for a consecutive case cohort that included external and local cases. Records were obtained for the Berichrom FXIII and HemosIL FXIII antigen assays (henceforth abbreviated as FXIII:Act and FXIII-A:Ag, respectively) and urea clot solubility tests [19].

As HRLMP safety authorities did not approve iodoacetamide use, FXIII:Act was performed by manual testing as recommended by the manufacturer, without an iodoacetamide blank, using Siemens Standard Human Plasma (referenced against a WHO standard by Siemens) as the calibrator, converting % activities to U/mL. The local reference interval (RI) of 0.60 to 1.69 U/mL was established in October 2000 using parametric analysis (mean ± 2SD) of *n* = 58 individual normal plasma samples. Results below 0.14 U/mL were reported as <0.14 U/mL based on our prior observation that FXIII:Act overestimated severely deficient samples [19].

Werfen FXIII-A:Ag (calibrated with Werfen calibration plasma, referenced to WHO standard 02/206 by Werfen, with % values converted to U/mL) was initially run on Stago instruments (STA-R, STA-R Evolution, Stago Canada Ltd) using a locally validated RI of 0.49 to 1.54 U/mL, established by nonparametric analysis of 69 individual normal plasmas in July 2011. From March 1, 2022, onward, the assay was run on Werfen ACL TOP 750 instruments, after verifying the manufacturer’s RI (0.75-1.55 U/mL) with 24 individual normal plasmas, EP Evaluator (EE; 12.1.0.18, Data Innovations), and acceptable EE comparisons of 19 diagnostic samples (including 4 from external quality assessment, EQA; FXIII-A:Ag levels: low: *n* = 15; normal: *n* = 4) tested in parallel on both analyzers. During the decade evaluated, HRLMP had acceptable performance for all FXIII assays in ECAT Foundation (External quality Control of diagnostic Assays and Tests) exercises.

Data gathered from laboratory records (identifiers anonymized after sorting by names, then date of birth, to identify unique cases) included patient sex (female or male), age (at first FXIII test), and FXIII test results. Electronic medical records for local FXIII-deficient patients assessed at Hamilton Health Sciences and St Joseph’s Healthcare Hamilton were retrospectively reviewed, along with records for 198 randomly selected local patients that were in the tested cohort but did not have FXIII deficiency. We extracted their reasons for testing; assessment locations (outpatient or inpatient, including ICU); if they had a congenital vs acquired bleeding problem; age when first tested for FXIII deficiency; age at the time of diagnosis of FXIII deficiency; types of bleeding; family history; transfusions and other treatments for bleeding and responses; relevant outcomes for FXIII-deficient patients such as response to treatments and in-hospital mortality; and relevant findings such as Clauss fibrinogen, evidence of other hemostatic disorders (eg, hemophilia and platelet function disorders), thrombocytopenia, consumptive coagulopathies, and disseminated intravascular coagulation (DIC), including ISTH DIC scores [20].

### 2.1 Statistical analysis

Descriptive statistics were used to evaluate test findings and patient characteristics, with counts and frequencies described for categorical variables (eg, sex) and medians and IQRs estimated for continuous variables (eg, age, FXIII:Act, and FXIII-A:Ag). For statistical analysis, FXIII results below RI limits were rounded up to the lowest reportable values (FXIII:Act: 0.14 U/mL; FXIII-A:Ag: 0.03 U/mL). Correlation coefficients were estimated to assess relationships between plasma FXIII:Act and FXIII-A:Ag and between plasma FXIII:Act and fibrinogen. Differences were assessed using the Pearson’s chi-squared test for categorical variables and the Wilcoxon rank sum test for continuous variables.

## 3 Results

### 3.1 Population evaluated

Table 1 summarizes the demographics and other details for the cohort evaluated, including ages (median: 21 years; range: 0-90 years; IQR: 2.5-48 years; 46% aged <18 years). Over the decade, 2171 samples from 1915 unique persons (59% females) had one or more FXIII tests. Among patients with quantitative FXIII results, 9.7% had a FXIII result below RI in one or more tests. Ages of FXIII-deficient patients were diverse (median: 36 years; range: 0-89 years; IQR: 7.5-62 years) and older than all evaluated patients (*P* < .0001). Local cases accounted for 37% of samples, 38% of all patients, and 30% of patients with FXIII deficiency by at least 1 quantitative test. Most local samples (62%) were from outpatients, and 30% of inpatient samples were from ICU.

**Table 1:** Demographics and other details on the patients assessed for factor XIII deficiency.

Characteristic	Result
Samples: number of external vs local Total number of samples, percentage external	1376 vs 815 total: 2191 samples, 63% external
Unique patients: number of external vs local Total number of patients, percentage external	1191 vs 724 total: 1915 patients, 62% external
Sex of patients External females vs males, percentage female Local females vs males, percentage female Total number of females vs males, percentage female	Unknown for *n* = 6 externals 695 vs 490, 58% 436 vs 288, 60% 1131 vs 778, 59%
% local cases tested while hospitalized % local hospitalized patients tested while in ICU % of local FXIII-deficient patients deceased at discharge	38% 30% 13% deceased (*n* = 6/48), all with acquired deficiency
Patient ages (at the time of first FXIII test)	Median: 21 y Range: 0-90 y (IQR: 2.5-48 y)
Percentage <18 y at the time of first FXIII test	46%
Percentage ≤1 y at the time of first FXIII test	22%
Percentage of unique patients with at least 1 quantitative FXIII result below the reference range (*n* = 168/1727 after excluding 4 patients with normal activity and a suspected false-positive low antigen)	9.7% (70% external)
Ages of patients with FXIII below the reference range in ≥1 quantitative test	Median: 17 y Range: 0-89 y (IQR: 0.5-55 y)

FXIII, factor XIII; ICU, intensive care unit.

### 3.2 FXIII assay findings

Table 2 summarizes FXIII and fibrinogen assay findings. Overall, a respective 14% and 9.7% of all evaluated samples and patients had FXIII-Act below adult RI for one or more samples, with varying deficiency severities (Table 2). FXIII-A:Ag was mainly used for the secondary investigation of FXIII deficiency, and 45% of samples and 41% of evaluated patients had low FXIII-A:Ag, with 19% of deficient samples containing minimal or undetectable levels (ie, ≤0.03 U/mL; Table 2). Samples with FXIII-A:Ag levels of ≤0.03 U/mL had FXIII:Act ranging from <0.14 to 0.20 U/mL, confirming that FXIII:Act overestimated severely deficient plasmas. Consistent with other reports [1,14,15], the urea clot solubility test detected only very severe FXIII deficiency (7/7 with undetectable FXIII-A:Ag) and failed to detect most (93%, 96/103) samples with low FXIII:Act (median FXIII:Act: 0.30 U/mL; range: ≤0.14-0.59 U/mL). Of additional concern, 17% (130/756) of urea clot solubility tests did not have an accompanying FXIII:Act or FXIII-A:Ag ordered or assessed to exclude FXIII deficiency.

**Table 2:** Summary of FXIII and fibrinogen test findings.

Test or parameter (adult RI) with numbers of evaluated samples (patients)	Details of deficient samples (patients) and findings	Percentage of all samples (patients) with FXIII deficiency	Details of nondeficient samples (patients) and findings
FXIII:Act, U/mL (0.60-1.69 U/mL) *n* = 1980 (1725)	*n* = 282 (167) median: 0.36 U/mL range: ≤0.14-0.59 (IQR: 0.16-0.43) U/mL	14% (9.7%) deficient: 22% (14%) with ≤0.14 U/mL 33% (17%) with <0.20 U/mL	*n* = 1723 (1558) median: 1.00 U/mL range: 0.60-2.99 (IQR: 0.84-1.19) U/mL
Werfen FXIII-A:Ag (0.49-1.54 U/mL on Stago instruments, 0.60-1.55 U/mL on Werfen instruments) *n* = 251 (170)	*n* = 113 (69) median: 0.25 U/mL range: ≤0.03-0.48^a^ (IQR: 0.04-0.36) U/mL	45% (41%) deficient: 19% (16%) with ≤ 0.03 U/mL	*n* = 138 (101) median: 0.87 U/mL range: 0.49-2.42 (IQR: 0.66-1.05) U/mL
Urea clot solubility test (abnormal: lysis) *n* = 754 (709)	*n* = 7 (7) with lysis	0.9% (1%) deficient, all with FXIII:Ag <0.03 U/mL	*n* = 747 (702) without lysis
Fibrinogen, g/L, for samples with FXIII:Act (1.6-4.2 g/L on Stago instruments, 2.0-3.9 g/L on Werfen instruments) *n* = 396 (394)	*n* = 15 (15)^b^, median: 1.3 g/L range: ≤0.2-1.8 (IQR: 0.9-1.5) g/L	3.8% (3.8%) deficient in fibrinogen 2% (2%) deficient in both fibrinogen and FXIII	*n* = 381 (379) median: 3.0 g/L range: 1.6-9.8 (IQR: 2.45-3.7) g/L

The numbers of samples evaluated for each test are shown, along with the findings for deficient and nondeficient samples based on local and published cutoffs.

FXIII, factor XIII; RI, reference interval.

a All abnormal Werfen FXIII-A:Ag results have been reported with the 0.49 U/mL RI limit for the assay run on Stago instruments.

b Fibrinogen deficiencies were based on age- and assay-specific RI.

Factor XIII inhibitor tests were requested for only 7 patients and 5 had detectable FXIII inhibitor levels (3 external, 2 internal; ages: 55-85 years; all with acquired FXIII deficiency) that was low titer (0.6-1.5 BU) for the 2 Hamilton patients and high titer for the 3 external cases (9-51 BU; first/last titers: 15.5 [single test], 35/12, and 51/22 BU). Most FXIII inhibitor patient samples (59%, 19/32) tested by both assays showed a “type 2” deficiency, with a low FXIII:Act (≤0.14-0.26 U/mL) but a normal FXIII-A:Ag, although a few (28%, 9/32, including 3/3 samples from 1 patient) had a “type 1” deficiency pattern (ie, similarly low FXIII by both assays) and 3/3 convalescent samples from the inhibitor patient whose FXIII deficiency resolved showed normal findings.

**Figure 1:**
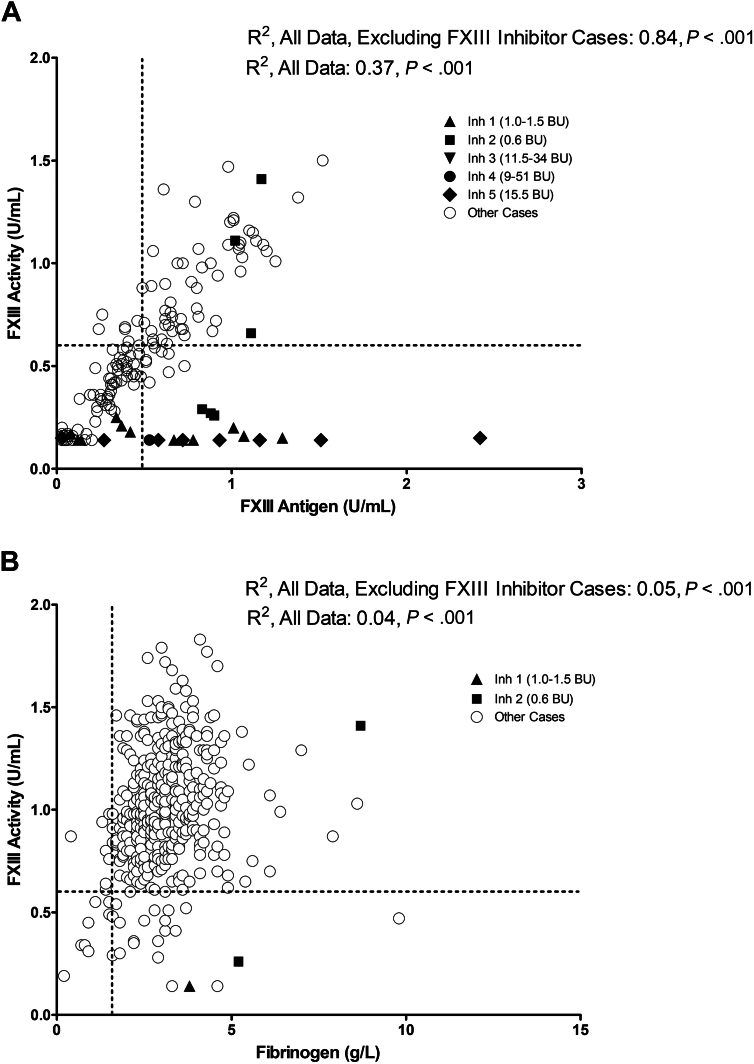
Relationship between factor XIII activity and Clauss fibrinogen levels and between factor XIII activity and factor XIIIA subunit antigen for patient samples. Results for samples that were tested for factor XIII activity (FXIII:Act) and FXIII-A subunit antigen (FXIII-A:Ag) (A) and for FXIII:Act and fibrinogen (B) are shown, along with the correlation coefficients. Solid symbols in both panels indicate samples from the 5 FXIII inhibitor cases (legend indicates the unique symbol for each inhibitor patient shown in the panels; data for other patients are shown with open circle symbols). Horizontal and vertical dashed lines indicate the lower limit of the RIs for the assays shown. For FXIII-A:Ag, most samples were tested when the lower RI limit for FXIII-A:Ag was 0.49 U/mL. For Clauss fibrinogen, most samples were tested when the lower RI limit was 1.6 g/L. FXIII, factor XIII; RI, reference interval.

In contrast to the disproportionate reduction in FXIII:Act relative to FXIII-A:Ag in many FXIII inhibitor patient samples (shown with solid unique symbols for each case in Figure 1A, with other cases shown with open symbols), most samples assessed by both assays (*n* = 187) had normal findings or similarly reduced FXIII activity and antigen. FXIII:Act and FXIII-A:Ag showed significant correlation (*R*^2^ = 0.37, *P* < .001) that increased (to *R*^2^ = 0.84, *P* < .001) when FXIII inhibitor patient samples were excluded.

Among 394 local patients with a Clauss fibrinogen evaluated at the same time as FXIII:Act (*n* = 396 samples; Figure 1B), 3.8% had a low fibrinogen and 2% had both a low fibrinogen and FXIII:Act either from a consumptive coagulopathy/DIC (*n* = 7; 4/7 from head injuries) or massive bleeding (*n* = 1). There was a weak but significant correlation between FXIII:Act and Clauss fibrinogen (*R*^2^ = 0.04 for all data, *P* < .001; *R*^2^ = 0.05 after excluding FXIII inhibitor patient samples, *P* < .001; Figure 1B).

### 3.3 Bleeding and laboratory findings for local FXIII-deficient patients

Among the 724 unique local patients in the cohort, 48 (6.6%) had FXIII:Act and/or FXIII:Ag below adult RI (81% assessed as inpatients, 46% of inpatients assessed in ICU). Their case information is summarized in Table 3, with details of bleeding listed in Table 4. A minority had an inherited deficiency (8.3%, 4/48) including 2 asymptomatic individuals tested because of a severely deficient sibling. Most (67%, 32/48) had FXIII evaluated after initial negative coagulation investigations. A minority had FXIII tests to assess replacement (12.5%, 6/48, 3 congenital, 3 acquired). Six of the 48 (12.5%; all acquired) patients died during the admission when the FXIII deficiency was diagnosed (details follow).

**Table 3:** Causes of factor XIII deficiency among local cases.

Cause of FXIII deficiency	Number of cases (numbers that died, proportion with follow-up tests that showed that the deficiency had resolved)	Age at diagnosis as median (range) in years	Nadir FXIII activity in U/mL, median (range) if applicable
Inherited (*n* = 4)			
Severe deficiency (undetectable FXIII-A:Ag)	3	0 (0-8)	<0.14
Asymptomatic carrier	1	26	0.55
Acquired due to FXIII autoantibodies (*n* = 2)			
Idiopathic, 1.0-1.5 BU inhibitor	1	73	<0.14
2^o^ to multiple myeloma, 0.6 BU inhibitor	1 (resolved)	56	0.26
Acquired due to a possible adverse reaction (*n* = 1) to Garcinia cambogia weight loss supplements	1 (resolved)	17	<0.14
Other acquired causes (*n* = 41)			
Consumptive coagulopathy	11 (2 deceased, 4/4 resolved)	3 (0.15-74)	0.45 (0.19-0.54)^a^
Major bleed	10 (3 deceased, 3/3 resolved)	55 (3-80)	0.39 (0.14-0.59)
Severe infection (*Streptococcal pneumoniae* meningitis, *n* = 1; culture-negative meningitis, *n* = 1; severe COVID-19, *n* = 2)	4 (1 deceased, 1/1 resolved)	42.5 (0.04-78)	0.49 (0.36-0.53)
Uncertain significance	16	55.5 (0-89)	0.41 (0.31-0.57)

FXIII, factor XIII.

a Nadir of FXIII-A:Ag was 0.47 U/mL for 1 patient who did not have FXIII:Act.

**Table 4:** Bleeding symptoms for locally evaluated patients with or without factor XIII deficiency.

Percentage (proportion) in each group with:	Low FXIII (inherited or acquired)	Normal FXIII	*P* value (if <.05)
Bleeding	92% (44/48)	88% (175/198)	
Bruising	13% (6/48)	20% (39/198)	
Wound bleeding	2.1% (1/48)	5% (10/198)	
Epistasis	10% (5/48)	16% (31/198)	
Oral bleeding	2.1% (1/48)	5% (10/198)	
Dental procedure bleeding	4.2% (2/48)	7.5% (15/198)	
Surgical procedure bleeding	6.3% (3/48)	7.5% (14/198)	
Heavy menstrual bleeding	12% (2/17)	33% (39/117)	
Postpartum bleeding	5.9% (1/17)	14% (17/120)	
Gastrointestinal bleeding	15% (7^a^/48)	4.5% (9/198)	.049
Muscle bleeds	0% (0/48)	1.5% (3/198)	
Joint bleeds	0% (0/48)	1% (2/198)	
Intracranial, subdural, and/or intraventricular bleeds	23% (11/48) (73% in children)	24% (47/198) (89% in children)	
Umbilical cord bleeds	6.3% (3/48)	0% (0/198)	.008
Other bleeds (retroperitoneal, adrenal, hematomas, nonmenstrual gynecologic, pancreatic, prostatic, pulmonary, aortic dissection, burn-related)	21% (10/48)	12% (24/198)	

Symptoms were retrospectively evaluated by medical record reviews for cases with low FXIII activity and for 198 randomly selected cases with normal FXIII activity. *P* values are shown for the symptoms that had a significantly different incidence for patients with or without factor XIII deficiency.

FXIII, factor XIII.

a All FXIII-deficient patients with gastrointestinal bleeding had an acquired FXIII deficiency.

Among the 4 patients with inherited FXIII deficiency, 3 had severe FXIII deficiency (undetectable FXIII-A:Ag prereplacement): one was diagnosed after an ICH at 8 years of age and another was diagnosed as a newborn after investigations of umbilical stump bleeding, which led to testing of his asymptomatic brother who also had severe FXIII deficiency. The fourth was an asymptomatic carrier (sister of the severe FXIII-deficient patient who had an ICH) who was not tested until she presented in labor with her first pregnancy (FXIII:Act: 0.55 U/mL; FXIII-A:Ag: 0.42 U/mL).

All other FXIII-deficient patients (44/48, 92%) had an acquired deficiency of varying severity (Table 3), often with bleeding (93%, 41/44). Among this group, 82% (9/11) with follow-up tests had normal convalescent FXIII levels (without replacement), confirming an acquired deficiency. Autoimmune FXIII deficiency due to an inhibitor (titers: 0.6-1.5 BU) accounted for 4.5% (2/44) of acquired FXIII deficiencies, and one resolved shortly after starting multiple myeloma treatment (a nadir FXIII:Act of 0.26 U/mL, normal FXIII-A:Ag on all samples; 0.6 BU inhibitor at presentation only). The other one was a previously reported patient [19] whose low titer FXIII inhibitor partially neutralized FXIII activity and accelerated FXIII clearance, leading to much lower FXIII:Act than FXIII-A:Ag for samples drawn shortly after replacement and very low FXIII:Act and FXIII-A:Ag on trough samples; he declined immunosuppressive therapy and was stably managed with weekly plasma-derived FXIII concentrate until death from unrelated causes 15 years later.

Another patient had confirmed acquired FXIII deficiency diagnosed at age 17 after presenting with acute posttonsillectomy bleeding requiring surgical intervention but not transfusions or FXIII replacement (acute levels, U/mL: FXIII:Act < 0.14-0.15, FXIII-A:Ag < 0.03-0.03; Table 3). She had taken a *Garcinia cambogia*–containing weight loss supplement, which can cause fulminant hepatic failure [21], for several weeks (liver function not assessed), and after stopping it, her bleeding resolved, with normal convalescent FXIII levels, assessed 6 months later.

The other 41 patients with acquired FXIII deficiency (44% diagnosed in ICU) had nadir FXIII:Act ranging from 0.14 to 0.59 U/mL (Table 3), with similar median nadirs (0.39-0.49 U/mL) for the groups with acquired FXIII deficiency due to with consumptive coagulopathies, severe infections, major bleeds, or a mild deficiency of uncertain significance. The 6 that died during their acute illness had acquired FXIII deficiency with major blood loss (*n* = 3, one with bowel ischemia), a consumptive coagulopathy (*n* = 2), or severe infection (*n* = 1). Those with consumptive coagulopathies included 6 children with acute brain injury/bleeds (2/6 with low fibrinogen), 3 adults with high ISTH DIC scores and/or multiorgan failure, an adult with massive bleeding and DIC from a type A aortic dissection, and an adult with massive bleeding and thrombosis of unknown cause. Among patients with acquired FXIII deficiency from massive bleeds (*n* = 10), one had previously undiagnosed Glanzmann thrombasthenia as the primary cause of bleeding. Among patients with nonsevere FXIII deficiencies of uncertain significance, some had other reasons or risk factors for bleeding, including vitamin K deficiency in a newborn with ecchymosis; severe hemophilia A in a child with a subdural hematoma (SDH) as their first hemophilia bleed; renal failure; thrombocytopenia; a liver disease coagulopathy; and local factors causing traumatic or gastrointestinal bleeds.

Sixty nine percentage (33/48) of the local FXIII-deficient patients received packed red cell transfusions for bleeding, 52% (25/48) received plasma, and 17% (8/48) received FXIII concentrate including 3 severe congenital deficient patients on prophylaxis; a carrier of FXIII deficiency treated prophylactically at the time of childbirth while awaiting FXIII results; an acquired FXIII-deficient patient with a low titer FXIII inhibitor and undetectable FXIII:Act on trough samples; and 3 additional acquired FXIII-deficient patients, including 1) a patient with chronic lymphocytic leukemia/small lymphocytic lymphoma and hypotensive shock (with bowel ischemia) from a severe, delayed bleed postrenal biopsy, requiring embolization, given FXIII replacement before a subsequent colectomy and a nephrectomy (nadir U/mL: FXIII:Act: <0.14, FXIII-A:Ag: 0.20; FXIII inhibitor not detected); 2) a patient with acquired FXIII deficiency of uncertain significance (nadir U/mL: FXIII:Act: 0.43; FXIII-A:Ag: 0.37) given FXIII prophylaxis before several surgeries; and 3) a patient who developed retroperitoneal bleeding after resolution of a COVID-19 consumptive coagulopathy, thrombocytopenia, and multiorgan failure (nadir U/mL: FXIII:Act: 0.36; FXIII-A:Ag: 0.25), given a single dose of FXIII concentrate that normalized his FXIII levels, with resolution of bleeding and sustained normal FXIII:Act upon recovery. The other patient with acquired FXIII deficiency during recovery from severe COVID-19 did not receive FXIII replacement as they had higher FXIII levels (U/mL: FXIII:Act: 0.49; FXIII-A:Ag: 0.32) and milder bleeding (wound oozing) managed by local measures.

Among acquired FXIII-deficient patients with evaluable FXIII:Act results (with respective patient numbers; median [range] in U/mL), nadir FXIII:Act was lower for those that did (*n* = 4; 0.25 [0.14-0.36]) vs those did not (*n* = 39; 0.46 [0.14-0.59]) receive FXIII concentrate (*P* < .04), but it was similar for those that did (*n* = 24; 0.45 [0.14-0.55]) vs those that did not receive plasma (*n* = 19; 0.46 [0.14-0.59]; *P* = .79). The newly diagnosed person with severe hemophilia A with low FXIII received FVIII concentrate (but not FXIII) for their SDH. The child with FXIII deficiency complicating a consumptive coagulopathy and purpura fulminans, with acquired protein C deficiency, received plasma and protein C concentrate.

Bleeding symptoms for the 48 local FXIII-deficient patients were compared to 198 randomly selected local cases whose tests excluded FXIII deficiency (Table 4). Briefly, 83% and 88% in each group, respectively, had bleeding, with similarity in symptoms, apart from more gastrointestinal (*P* = .049) and umbilical cord (*P* = .008) bleeding for FXIII-deficient patients (Table 4). Umbilical cord bleeding led to testing of 3 newborns and revealed that 1 had severe FXIII deficiency, whereas the other 2 had a mild FXIII deficiency of uncertain significance. The most common type of bleeding was ICH, SDH, and/or intraventricular bleeding, which was present in 23% to 24% of patients with or without FXIII deficiency, mainly in children (73%-89%; Table 4).

## 4 Discussion

FXIII deficiency is a challenging-to-diagnose disorder, with inherited and acquired causes that affect persons of diverse ages. This 10-year retrospective review was undertaken to gather unbiased information on the causes and severity of FXIII deficiency and the types of bleeding seen in congenital and acquired FXIII-deficient patients compared to representative local patients excluded of having FXIII deficiency. We also evaluated 10 years of HRLMP clinical diagnostic sample results for a cohort of consecutively tested, pediatric and adult cases, finding low FXIII in 14% and 9.7% of 2171 samples and 1915 patients, with local cases accounting for 38% of samples and 30% of FXIII deficiencies. FXIII activity and antigen level showed strong correlation, except for FXIII inhibitor cases, and both assays helped diagnose and characterize FXIII deficiencies. Importantly, among local patients, acquired FXIII deficiency was ∼10-fold more common than inherited FXIII deficiency, and the severities and causes of acquired deficiencies varied and included deficiencies of uncertain significance (36%), deficiencies from consumptive coagulopathies (25%), major blood loss (23%), severe infection (9%), and, infrequently, deficiencies from an adverse reaction (2.3%) or FXIII inhibitors (4.5%). Most (92%) FXIII-deficient patients had bleeding, although the primary cause for several of them was previously undiagnosed severe hemophilia A or Glanzmann thrombasthenia, and some had other contributors to bleeding, such as vitamin K deficiency, thrombocytopenia, liver disease, renal failure, or a local lesion. These observations illustrate the real-life challenges of assessing patients with low FXIII.

The respective yields of FXIII:Act and FXIII-A:Ag tests among evaluated patients were a reflection of the diagnostic approach taken, in which FXIII:Act (13% abnormally low) was used to screen for FXIII deficiency, and FXIII-A:Ag (44% abnormally low) was often used to further characterize abnormal FXIII:Act results, including if FXIII was near limits of detection (<0.14 U/mL for FXIII:Act vs ≤0.03 U/mL for FXIII-A:Ag) of FXIII:Act run without an iodoacetamide blank. A minority of diagnostic laboratories measure FXIII:Act using an iodoacetamide blank [15,22]. Some laboratories do not permit iodoacetamide use as it can cause acute oral toxicity, aquatic life hazards, category 1 (most severe) skin irritation, and skin and respiratory sensitization, which require protective measures [23]. Some samples with FXIII-A:Ag ≤ 0.03 U/mL had 0.15-0.20 U/mL FXIII:Act, supporting the 0.20 U/mL concentration used by some laboratories as the lower limit of detection for the unmodified Berichrom FXIII assay.[15] Some patients with low FXIII:Act had normal or much higher FXIII:Ag due to a FXIII inhibitor detectable by a Bethesda assay modification of the FXIII:Act assay. These observations highlight the importance of screening for low FXIII with a quantitative activity assay and further evaluating deficient samples to assess FXIII-A:Ag, which is more cost-effective than running both tests on all samples.

In our study, the urea clot solubility test was positive in <1% of samples, and it missed most (93%) cases with low FXIII:Act, which supports expert recommendations to assess FXIII deficiency by quantitative assays rather than clot solubility tests [1]. While we had historically used the urea clot solubility test to determine if samples with FXIII:Act ≤ 0.14 U/mL had any detectable FXIII, FXIII-A:Ag proved much more informative. In almost one fifth of the cases, a urea clot solubility test was ordered without a FXIII:Act, against expert recommendations [1], which led us to take corrective actions (eg, recommending that a FXIII:Act be added on to external samples, substituting FXIII:Act as the screen for internal samples, and initiating plans to discontinue the urea clot solubility test).

FXIII inhibitor testing, though rarely requested, was positive for 2 local and 3 external patients with acquired FXIII deficiency (aged 55 to 85 years) with many inhibitor patient samples showing a “type 2” deficiency pattern with a disproportionately low FXIII:Act compared to FXIII-A:Ag, unlike other acquired and inherited FXIII deficiencies. In contrast, samples without inhibitors showed strong correlation between FXIII-A:Ag and FXIII:Act (*R*^2^ = 0.84). Samples with “type 2” FXIII deficiency patterns should raise suspicion about an inhibitor and they represent a “blind spot” for laboratories that assess FXIII-A:Ag but not FXIII:Act, which could compromise the diagnosis of acquired FXIII deficiency due to FXIII autoantibodies, unless the inhibitor accelerates FXIII clearance.

Untreated congenital FXIII deficiency has high morbidity and mortality [24]. The severe congenital FXIII-deficient patients at our center were doing well on replacement to limit bleeding. In our local cohort, which included 8% congenital and 92% acquired FXIII-deficient patients, most (83%, 41/48) FXIII-deficient patients had bleeding, which had triggered investigations and had required packed red blood cell transfusions in 69% of patients, with 50% receiving plasma and 15% receiving FXIII concentrate. The predominance of acquired FXIII-deficient cases (with only 4.5% from inhibitors) in our cohort may be surprising as much of the hematology literature on FXIII deficiency is focused on inherited and autoimmune causes [[25], [26], [27]]. One challenge we faced was the lack of an accepted framework to classify acquired FXIII deficiencies without an inhibitor, which were often associated with a consumptive coagulopathy, major blood loss (sometimes from significant local and/or underlying hemostatic defects), severe infection (including COVID-19, which lowers FXIII [28], probably due to consumption), or a defect of uncertain significance, which had similar median nadir FXIII levels. Not surprisingly, the patients in our cohort who received FXIII concentrate had significantly lower FXIII levels than other FXIII-deficient patients. While it is tempting to use information on bleeding at different FXIII:Act nadir levels to propose a level of FXIII that merits replacement in acquired deficiencies, in reality, the clinical decisions on treatment were complex and influenced by the severity of bleeding, need for surgical prophylaxis, and if there were other causes/contributors to bleeding, including a previously undiagnosed primary bleeding disorder (such as Glanzmann thrombasthenia or severe hemophilia A), vitamin K deficiency, a consumptive coagulopathy with both low fibrinogen and FXIII, thrombocytopenia, liver disease, renal failure, or an identified local cause of bleeding. Information on inherited FXIII deficiency suggests that FXIII levels of ∼0.31 U/mL, perhaps up to ∼0.51 U/mL [8] (the upper limit of the 95% CI estimate), are needed for hemostasis. The patient in our cohort who had a retroperitoneal bleed while recovering from severe COVID-19, with nadir FXIII:Act and FXIII-A:Ag of 0.36 and 0.26 U/mL, respectively, and responded to FXIII replacement suggests that the FXIII levels needed for hemostasis are likely similar for congenital and acquired deficiencies.

The similarity in bleeding symptoms reported by the FXIII-deficient and nondeficient patients in our cohort is quite interesting and likely reflects that FXIII tests were ordered for patients with bleeding that a clinician suspected could reflect FXIII deficiency. Intracranial bleeding was the most common type of bleeding in both, and many were children with bleeds following head trauma. However, only patients with FXIII deficiency had umbilical bleeding. FXIII-deficient patients (all acquired) had more gastrointestinal bleeding than those without FXIII deficiency, which is interesting as among patients with gastrointestinal bleeding, acquired FXIII deficiency is associated with higher transfusion needs [29].

In its heterotetramic proenzymatic form, plasma FXIII circulates bound to fibrinogen [3], and we found a weak but significant correlation relationships between plasma Clauss fibrinogen and FXIII levels (*R*^2^ = 0.05 after excluding inhibitor patients; *P* < .0001) among local patients. Importantly, we also identified that some patients had combined fibrinogen and FXIII deficiencies from a major bleed or consumptive coagulopathy. Interestingly, FXIII and fibrinogen levels have previously been correlated in patients with venous malformations, which can cause consumptive coagulopathies [30]. The longer half-life of FXIII compared to fibrinogen (11-14 days vs 72-120 hours) [31,32] suggests that recovery from acquired deficiencies could be slower for FXIII, as observed for a COVID-19 patient who had retroperitoneal bleeding from acquired FXIII after his fibrinogen level had normalized. In trauma-induced coagulopathies, there has been emphasis on evaluating and replacing fibrinogen [33]. FXIII in cryoprecipitate and fibrinogen concentrates [34] may help correct accompanying FXIII deficits in such patients given the correlations between FXIII and fibrinogen levels and between FXIII and thromboelastometry estimates of fibrin contributions to clot firmness [4]. There could be opportunities to optimize care, as treating bleeding trauma patients with FXIII concentrate has been reported to reduce needs for fresh frozen plasma and packed red blood cell transfusions [13,35].

Several limitations of this study are inherent to its retrospective design. We did not have clinical context information on external patients and test ordering practices varied. For local patients, the decision to test was not standardized, and bleeding was not uniformly assessed and some details were not recorded (eg, times between hemostatic challenges and bleeding onset, as FXIII deficiency causes delayed bleeding). Also, we did not estimate FXIII:Act with an iodoacetamide blank. Nonetheless, our study has significant strengths that include the high potential for external validity due to the large number of evaluated samples/patients, the consecutive sample and case cohort design to limit bias, the similarity in the proportion of all patients and local patients with FXIII deficiency, the varying severities of FXIII deficiencies among all patients and local cases, the detailed analysis of local patients including bleeding symptoms and other conditions and contributors to bleeding, the comparison of FXIII:Act and FXIII-A:Ag using the most commonly used FXIII activity and antigen assays, and the comparison of FXIII:Act and Clauss fibrinogen findings. The strong correlation between FXIII:Act and FXIII-A:Ag was expected and the weak correlation between FXIII:Act and fibrinogen deficiency was interesting, as were the dual deficiencies in some patients with massive bleeding or consumptive coagulopathies.

In summary, the diagnosis of FXIII deficiency remains challenging. Physicians and laboratories need to be aware of the much higher prevalence of acquired compared to congenital FXIII deficiency, and acquired deficiency can result from inhibitors, consumptive coagulopathies, major bleeds, severe infections, or deficiencies of uncertain clinical significance and there may be other reasons for bleeding. We support simplified FXIII testing algorithms [7] and recommend that laboratories and clinicians consider the following:

1) If only FXIII-A:Ag is available to quantify FXIII, consider sending samples to another site to obtain a quantitative FXIII:Act, and exclude “type 2” deficiencies that are typical of FXIII inhibitors.
2) If FXIII:Act is evaluated, deficient samples should be tested for FXIII-A:Ag to further characterize the deficiency, particularly at diagnosis.
3) If autoimmune FXIII deficiency from an inhibitor is suspected, quantify both FXIII:Act and FXIII-A:Ag as disproportionately reduced activity is more common than a “type 1” deficiency with acquired inhibitors, and test FXIII-deficient samples for neutralizing antibodies by a Bethesda assay modification of FXIII:Act. Monitor responses to assess for rapid clearance and/or neutralization of infused FXIII that could suggest an inhibitor complicating an inherited FXIII deficiency or causing acquired FXIII deficiency.
4) Quantify Clauss fibrinogen if an acquired deficiency from consumption and/or major blood loss is suspected, and determine the ISTH DIC score [20] if consumption is the likely cause.
5) Consider follow-up testing to verify that a suspected acquired FXIII deficiency resolves.
6) If quantitative FXIII assays are available, discontinue use of FXIII clot solubility assays, as quantitative assays are superior for diagnosing, characterizing, and monitoring FXIII deficiency.
7) Consider other causes and contributors to bleeding in patients with mild acquired FXIII deficiencies (nadirs > 0.31 U/mL), given uncertainties about bleeding risks for FXIII levels above ∼0.31 to 0.51 U/mL.
8) Consider shared decision-making for managing acquired FXIII deficiencies, with consideration of the patient’s FXIII levels, the FXIII levels associated with bleeding in congenital FXIII deficiency, and other causes/risks/contributors to bleeding.
